# Investigation of Q Angle and Related Factors in Osteoporosis Patients

**DOI:** 10.5152/eurasianjmed.2025.25946

**Published:** 2025-09-03

**Authors:** Nurseda Başgün, Muhammet Şahin Elbastı, Songül Bağlan Yentü

**Affiliations:** 1 Department of Physical Therapy and Rehabilitation, Fırat University Faculty of Health Sciences, Elazığ, Türkiye; 2 Department of Physical Therapy and Rehabilitation, Elazığ Medical Hospital, Elazığ, Türkiye

**Keywords:** Osteoarthritis, osteopenia, osteoporosis, Q angle

## Abstract

**Background:**

The aim of this study was to examine the Q angle in patients diagnosed with osteoporosis, osteopenia, and osteoarthritis and to compare the groups with each other.

**Methods:**

This study included 22 female patients diagnosed with osteoporosis, 22 female patients diagnosed with osteopenia, and 22 female patients diagnosed with osteoarthritis, aged 40-65 years. Q angle, pain, lower extremity muscle strength, foot posture, and pes planus were assessed using a goniometer, Visual Analog Scale (VAS), Medical Research Council (MRC) Scale, Foot Posture Index (FPI), and Navicular Drop Test (NDT), respectively.

**Results:**

There was a statistical difference in both right and left Q angle in osteoporosis compared to osteoarthritis (*P* = .009, *P* = .002, respectively). There was no statistically significant difference in both right and left Q angle between osteoporosis and osteopenia (*P* = .730, *P* = .491, respectively), and osteopenia and osteoarthritis (*P* = .022, *P* = .017, respectively). However, a statistically significant difference was found between osteoporosis and osteoarthritis in the right (*P* = .009) and left (*P* = .002) sides. In addition, a significant negative correlation was found between Q angle and FPI and NDT in all patients in this study (*P* < .01).

**Conclusion:**

Patients with osteoporosis were found to have a higher Q angle than osteoarthritis and osteopenia patients. In addition, foot posture and pes planus were found to be associated with the Q angle. Osteoporosis patients may have a tendency to valgum.

**Main Points**

- Patients with osteoporosis were found to have a higher Q angle than osteoarthritis and osteopenia patients.
- It was concluded that the Q angle was associated with foot posture and navicular drop test in patients with osteoporosis, osteopenia, and osteoarthritis patients. Therefore, osteoporosis patients may have a tendency to valgum.
- Evaluation of the relationship of Q angle with muscle strength and hip anteversion angle should be investigated.

## Introduction

The Q angle, an important parameter in physiotherapy, plays a significant role in understanding biomechanics and addressing musculoskeletal problems. The Q angle is the angle at which 2 lines—1 from the patellar center (PC) to the anterior superior iliac spine and the other from the PC to the tibial tuberosity—intersect.^1^ The Q angle is an anatomical measurement used to assess the alignment of the lower extremities, especially the knee joint. When the data obtained from many studies in the literature were combined, it was reported that Q angle values between 10° and 14° for men and 14.5° and 17° for women were normal.^2^^,^^3^

Physiotherapists often use the Q angle to identify biomechanical problems that may contribute to musculoskeletal issues. It has been reported that the Q angle changes in some diseases.^4^ An altered Q angle can lead to various clinical changes.^5^ A higher Q angle has been associated with increased lateral forces on the patella, potentially leading to conditions such as patellofemoral pain syndrome and patellar instability. During assessments, physiotherapists consider factors such as pelvic tilt, muscle imbalances, and joint flexibility to gain a comprehensive understanding of the patient’s biomechanics.^6^^,^^7^

Osteopenia (OPN) and osteoporosis (OP) are considered to be diseases on the same spectrum. Osteopenia is a disease with milder symptoms than OP. In various literature reports, OPN is considered the precursor of OP.^8^ Reduced bone density and bone tissue degradation are hallmarks of OP, a bone disease that raises the risk of fractures. The development of OP is influenced by a number of factors, such as age, gender, and hormonal and genetic changes. A decline in estrogen levels, which is essential for preserving bone density, puts women at greater risk, particularly those who have gone through menopause.^9^^-^^11^ Pain due to vertebral fractures, shortening in height, dyspeptic complaints due to shortening in the pubis-vertebral distance, decrease in lung expansion and cardiac functions, and an increase in mortality may develop, while walking problems, prolonged disability, and an increase in mortality may be observed due to hip fractures in OP. The most commonly involved regions in OP are the vertebrae and hips. It is thought that involvement in these regions may affect lower extremity biomechanics.^12^ While complications following vertebral and hip fractures in patients with OP have been examined in the literature,^13^ no study on lower extremity biomechanics has been found. To the best of the authors’ knowledge, there is no study evaluating the Q angle in patients with OP, which may provide insight into lower extremity malalignment.

The most prevalent characteristics of osteoarthritis (OA) include subchondral bone sclerosis, synovial inflammation, osteophyte production, and joint-wide involvement with loss of cartilage structure. This results from variations in how loads are distributed over the knee’s medial and lateral compartments. Studies on the Q angle in OA patients have shown that the affected area and severity of the disease affect the findings. There may be varus or valgus knees, a change in the load distribution between the lateral and medial compartments of the joint, and a rise or fall in the Q angle.^14^

To the best of the authors’ knowledge, there are studies assessing Q angle in patients with OA, but none assessing Q angle in patients with OP or OPN. The purpose of this study was to assess the Q angle in people with OP, OPN, and OA and ascertain how it related to the evaluation of pes planus, foot posture, and muscular strength.

## Materials and Methods

This study comprised 22 female patients with a diagnosis of OP, OPN, or OA who were between the ages of 40 and 65. The individuals who volunteered to take part in the study gave their informed consent. Before the study began, approval from the Fırat University Ethics Committee was acquired (Approval no: 2024/07-33; Date: 09.05.2024). Age, height, weight, body mass index, medical history, and last name were among the demographic data of the participants.

The G*Power Ver. 3.1.9.4 tool was used to determine the study’s sample size and power requirements. A minimum of 22 participants should be included in each group, according to the power analysis, which was based on the results of eta squared (η²) = 0.14 with a risk of α = 0.05, 1 − α = 0.80 accuracy rate condition, and effect size 0.40. The actual power was 0.81. There were 66 participants after the study was finished.

### Participants and Study Design

The study was designed as a cross-sectional clinical study and included female volunteers between the ages of 40 and 65 who had been diagnosed with OP and OPN using the World Health Organization classification system and had lumbar vertebrae and femoral neck T scores of <−2.5 SD for OP patients and between −1 and −2.5 SD for OPN patients. They also had to have had a dual-energy X-ray absorptiometry (DEXA) device at least a year prior. The inclusion criteria for the OA group were specified as those affected medial components and included in the grade 1 and grade 2 groups. Patellofemoral abnormalities during examination, acute traumatic patellofemoral dislocations, surgical revision around the knee, history of lower extremity surgery, severe trauma, and having neurological, orthopedic, or rheumatologic disease affecting the lower extremities were the exclusion criteria for the OP, OPN, and OA groups (paresis, paralysis, rheumatoid arthritis, etc.).

### Outcome Measurements

#### Q Angle Measurement

The Q angle was measured with a goniometer. The measurement was performed while the person was standing. In standing measurements, the knees should be in extension, and equal load should be given to both lower extremities. The person was asked to remain motionless until the end of the measurement process, with the thigh area unclothed, standing upright, quadriceps muscle in a relaxed position, and feet in a neutral position. The measurements were repeated 3 times and the average of the 3 measurements was taken. The Q angle measurements were performed by the same person in the same environment, and a standardized situation was established. Measurements were performed on both lower extremities.^15^

#### Pain Assessment

Pain was assessed using the Visual Analog Scale (VAS). A chart that reads “I have no pain” on one end and “very severe pain” on the other is used to perform this measurement. On this line, the patient indicates their present state of health. The distance between the patient’s designated location and the pain-free location reveals the patient’s level of pain.^16^

#### Muscle Strength Assessment

The Medical Research Council (MRC) Scale was used to measure the strength of the muscles in the lower extremities. Muscle tone and resistance to gravity are used to grade muscle strength, which ranges from 0 to 5.^17^

#### Foot Posture

The FPI (Foot Posture Index) was assessed. The FPI is a simple, valid, and trustworthy technique that assesses the foot in a multifaceted and thorough way and yields a single numerical answer that indicates the degree of pronation and supination of the foot.^18^

#### Pes Planus Evaluation

It was evaluated with the Navicular Drop Test (NDT). The NDT is a test obtained by subtracting the navicular height measured in the standing position with weight on the foot from the navicular height measured in the sitting position without weight on the foot.^19^

### Statistical Analysis

Analysis of the data gathered for the study was done statistically using SPSS 22.0 software (IBM SPSS Corp.; Armonk, NY, USA). Numbers, percentages, and medians were used to display descriptive data [Q1;Q3]. The Kolmogorov-Smirnov test was used to examine the normality distribution. Numerical data that did not meet the normal distribution were compared using Kruskal–Wallis tests. In intergroup comparisons, if *P* < .05 after the Kruskal–Wallis test, the significance was checked according to the new *P* value (*P* < .017) depending on the number of groups, with Bonferroni correction, to reveal which group/groups the difference originated from.

## Results

The demographics of the OA, OPN, and OP groups were compiled in Table 1. There was no significant difference between the groups in terms of demographic data (age: *P* = .729, height: *P* = .540, weight: *P* = .758, BMI: *P* = .971). Based on FPI and NDT measures, Table 2 summarized the number and percentage of patients in each group were summarized. The VAS, FPI, NDT, and Q angle measurements between the groups were compiled in Table 3. Only the Q angle differed statistically across the groups (*P* = .017 for the right extremity and *P* = .005 for the left). In the post hoc test to determine which group or groups this difference originated from, a statistical difference was found in both the right and left Q angle when the OP group was compared with the OA group (*P* = .009, *P* = .002, respectively). There was no statistically significant difference in both right and left Q angle between OP-OPN (*P* = .730, *P* = .491, respectively) and OPN-OA (*P* = .022, *P* = .017, respectively) groups. However, a statistically significant difference was found between OP-OA groups (*P* = .009 for the right extremity and *P* = .002 for the left extremity). Table 4 provided a summary of the three groups’ muscular strength comparison. There was no difference in muscle strength between the three groups (*P* > .05). In every patient group, there was a negative connection between Q angle left and right readings and both FPI and NDT (Table 5 and 6). A negative correlation was found between Q angle left and right measurements and NDT and FPI in all patient groups (Table 5 and 6).

## Discussion

The purpose of this study was to assess the Q angle in patients with OP, OPN, and OA and investigate its correlation with pes planus and foot position. The study’s findings demonstrated that OP and OA patients had substantially differing Q angles. In addition, the Q angle was associated with foot posture and navicular drop test in all groups. Although there are controversial results in OA patients, it is known that the Q angle decreases in grade 1 and 2 patients. The difference between OP and OA may stem from the disease severity of OA. It was also observed that the Q angle was higher in OP patients compared to other patients. This suggested that these patients may be predisposed to valgus. Therefore, the addition of foot and knee evaluation in OP patients may provide information about lower extremity alignment.

Q angle is a parameter used to evaluate the biomechanical status of the patellofemoral joint and the smoothness of the lower extremity alignment. In addition, it is also used to define the relationship between sports injuries and structural factors and as a preliminary indicator of predisposition to sports injuries.^20^ It was concluded that Q angle measurements in both lower extremities were significantly different in the OP group compared to the OA group in this study. Q angle measurements in OA patients differ according to the severity of the disease. It was reported that the Q angle increased in patients with grade 3 and 4 OA.^14^ On the other hand, according to the study of Çiftçi and Kurtoğlu, it was found that the Q angle decreased in OA patients and caused genu varum in these individuals.^21^ Genu varum deformity is frequently seen due to the fact that the medial joint space is more affected in patients with gonarthrosis. In addition, it has been demonstrated that an increase in radiographic stage grade in patients with knee OA leads to a decrease in femoral cartilage thickness, and this decrease leads to an increase in limitation in physical functions and disability. The Q angle has also been found to be significantly correlated with radiologic stage severity in the same direction. A high or low Q angle throws off the knee joint’s alignment. By accelerating the deterioration of knee joint components, misalignment may have a detrimental effect on the osteoarthritis process.^22^ Whether the Q angle was high or low, it was determined that the knee joint alignment was disturbed, which could have a detrimental effect on the OA process and accelerate the deterioration of the knee joint components. As a result, the Q angle needs to be taken into account for knee rehabilitation.

Deviation of the Q angle in patients with knee OA is thought to affect the distribution and intensity of cartilage stresses.^23^ A significant correlation between the deviation (increase or decrease) of the Q angle from physiologic values and the progression of radiologic and articular cartilage semiquantitative findings was found in female patients with knee OA. The findings also emphasize the importance of Q angle measurement in the early diagnosis and follow-up of knee OA.^24^ According to the results of this study, Q values of all patients were within normal limits. However, the fact that the Q values of the patients with OA were at the lower limit may be due to the fact that the patients who participated in this study had medial involvement and therefore may have genu varum. This study demonstrated that individuals with OP and OPN also had normal Q angle values; however, these values were higher than those of OA patients. It can be concluded that OP is less likely to trigger a degenerative process such as OA.

The strength of the muscles around the hip, knee, and ankle in OP, OPN, and OA patients was evaluated in this study. It was determined that the groups did not differ significantly from one another. In addition, it was observed that all participants had good muscle strength. Evaluation with manual muscle testing may have prevented the measurement of small differences. It is known that the muscles around the knee and hip are affected in patients with OA. However, OA patients who participated in this study may have good muscle strength because they were grade 1 and 2. Again, the good muscle strength of all patients who participated in this study may be related to the normal values of the Q angle.^25^ Osteoporosis has a serious effect on the biomechanics of the musculoskeletal system.^26^ It has been determined that the difference in quadriceps muscle size and strength may cause a difference in the Q angle. There is an inverse relationship between quadriceps muscle strength and the Q angle. Especially Q angle values below 10º provide more effective traction for the quadriceps muscle. Karabulut et al^2^ investigated the relationship between the Q angle and muscle atrophy. It could not be defined whether increased angle values were due to muscle atrophy or whether muscle atrophy was due to increased Q angle. Because of the pulling impact of the quadriceps muscle, You et al^27^ discovered that sports involving a lot of quadriceps strength training are linked to lower Q angles. However, due to interconnections between anatomical structures, the traction dynamics surrounding the knee joint are a complex aspect. Although the association between the Q angle and quadriceps muscle strength has been documented, it may differ depending on the sport.^27^ Different results can be obtained by using objective assessment methods, and small differences can be revealed.

The Q angle was found to be a value often taken as a reference for the alignment of the lower extremities.^28^ An excellent correlation between the Q angle and NDT and FPI tests was found in OP and OA patients in this study. This result demonstrated that foot posture and medial arch drop may be related to the Q angle. Q angle decreases with increasing medial arch drop. It is known that pes planus and foot pronation are seen in OA patients with medial component involvement. It was also determined that the Q angle affected the foot posture in OP patients, and the foot was mostly in the pronation position according to this study. While the loading line positioned medially in the pronated foot raises the adduction moment in the knee joint, which can only be decreased with a larger Q angle, the loading line adjusted laterally in the supinated foot minimizes the adduction moment. Navicular drop measurements were demonstrated among the parameters thought to have an effect on the Q angle. A significant relationship was found between increased navicular drop test results and the Q angle in parallel with this study.^29^ Navicular height was concluded to be the most important contributing factor for the Q angle, which can change the plantar pressure distribution by aligning the foot posture to supination or pronation. It was also reported that increased load on the lateral part of the forefoot was associated with a decrease in the Q angle.^30^ A positive and moderately significant relationship was found between the Q angle, hip-knee-ankle angle, mechanical lateral distal femoral angle, right femoral Q angle, right ankle tilt angle values, and K-L stages. In addition, it was emphasized that changes in these parameters should be paid attention to in the follow-up of patients with knee OA.^31^

In this investigation, there was no discernible variation in the groups’ BMIs or Q angles. BMI values of the patients participating in this study were close to each other, and the number of obese patients was low. The lack of a significant relationship with the Q angle may be due to the lack of patients with different indices. According to Erden et al,^15^ the Q angle and BMI, waist circumference, and hip circumference were positively and moderately significantly correlated.

It was also suggested that decreased lower extremity strength due to low physical activity levels in female office workers and increased BMI, waist, and hip circumference may cause an increase in the Q angle.^15^ Different results may be obtained in studies conducted with patients with different BMI values.

### Study Limitations

The study includes evaluations of OP, OPN, and OA patients. The absence of a control group including healthy individuals is a limitation of the study. Another limitation is that this study was performed only on a female patient group and not on males. Not measuring femoral anteversion angle can be seen as a limitation. In addition, sensitivity is not high because manual muscle testing was performed.

## Conclusion

According to the study’s findings, patients with OP and OPN had Q angles that were higher than those of patients with OA but still within normal bounds. This may be due to the possibility of genu varum in individuals with OA. Additionally, the lower Q angles of patients with OPN may be interpreted as more valgus in patients with OP. It was observed that the Q angle was associated with foot posture and navicular drop test in OP, OPN, and OA patients. Q angle is an important parameter in terms of providing information about the lower extremity alignment status. Clinical evaluation of the Q angle in OP patients may be important for providing information about valgum deformity and foot posture in these patients. Further studies should include an evaluation of the relationship of Q angle with muscle strength and hip anteversion angle.

## Figures and Tables

**Table 1.** Comparison of Demographic Data Between Osteoporosis, Osteopenia and Osteoarthritis Groups

	**OP Group (n = 22)** Median [Q1;Q3]	**OPN Group (n = 22)** Median [Q1;Q3]	**OA Group (n = 22)** Median [Q1;Q3]	***P***
Age (years)	58.00 [54.50; 61.25]	55.00 [52.00; 61.00]	55.50 [53.00; 62.00]	.729
Height (m)	1.60 [1.55; 1.62]	1.58 [1.52; 1.62]	1.60 [1.55; 1.63]	.540
Weight (kg)	72.00 [63.75; 83.50]	71.50 [62.50; 79.25]	71.00 [66.00; 76.25]	.758
BMI (kg/m^2^)	28.93 [24.60; 32.11]	28.62 [25.39; 30.57]	28.15 [25.68; 30.94]	.971

BMI, body mass index; kg, kilograms; m, meter; m^2^, square meter, OA, osteoarthritis; OP, osteoporosis; OPN, osteopenia.

**Table 2.** Distribution of Foot Posture Index and Navicular Drop Test in Osteoporosis, Osteopenia and Osteoarthritis Groups

		OP Group (n = 22) n (%)	OPN Group (n = 22) n (%)	OA Group (n = 22) n (%)
FPI-R	Normal	9 (40.9)	15 (68.2)	18 (81.8)
	Pronated	7 (31.8)	2 (9.1)	1 (4.5)
	Highly pronated	0 (0)	0 (0)	0 (0)
	Supinated	5 (22.7)	5 (22.7)	3 (13.6)
	Highly supinated	1 (4.5)	0 (0)	0 (0)
FPI-L	Normal	8 (36.4)	13 (59.1)	16 (72.7)
	Pronated	7 (31.8)	4 (18.2)	2 (9.1)
	Highly pronated	0 (0)	0 (0)	0 (0)
	Supinated	6 (27.3)	5 (22.7)	4 (18.2)
	Highly supinated	1 (4.5)	0 (0)	0 (0)
NDT-R	Normal	14 (63.6)	19 (86.4)	21 (95.5)
	Pronated	5 (22.7)	1 (4.5)	0 (0)
	Supinated	3 (13.6)	2 (9.1)	1 (4.5)
NDT-L	Normal	14 (63.6)	21 (95.5)	21 (95.5)
	Pronated	4 (18.2)	1 (4.5)	0 (0)
	Supinated	4 (18.2)	0 (0)	1 (4.5)

FPI, Foot Postur Index; L, left; NDT, Navicular Drop Test; OA, osteoarthritis; OP, osteoporosis; OPN, osteopenia; R, right.

**Table 3.** Intergroup Visual Analog Scale and Functional Comparison of Measurements

	OP Group (n = 22) Median [Q1;Q3]	OPN Group (n = 22) Median [Q1;Q3]	OA Group (n = 22) Median [Q1;Q3]	*P*	*P*_1-2_	*P*_1-3_	*P*_2-3_
VAS	4.00 [3.75; 6.00]	4.00 [3.00; 5.00]	4.00 [3.00; 6.00]	.612			
FPI-R	3.50 [-2.00; 8.00]	4.00 [-0.50; 5.00]	4.00 [2.00; 4.00]	.994			
FPI-L	3.00 [-3.00; 7.25]	3.50 [-0.50; 5.00]	3.00 [2.25; 4.25]	.995			
NDT-R	8.00 [5.75; 9.25]	6.50 [5.00; 8.25]	7.00 [6.00; 7.00]	.193			
NDT-L	7.50 [5.00; 9.00]	6.50 [5.75; 9.00]	6.00 [6.00; 8.00]	.594			
Q Angle-R	15.00 [12.00; 16.00]	13.50 [12.75; 16.00]	12.00 [11.00; 14.00]	**.017**	.730	**.009**	.022
Q Angle-L	15.50 [13,00; 17.00]	14.00 [12.00; 16.00]	12.00 [11.00; 14.00]	**.005**	.491	**.002**	.017

FPI, Foot Posture Index; L, left; NDT, Navicular Drop Test; OA, osteoarthritis; OP, osteoporosis; OPN, osteopenia; p 1-2, comparison of OP-OPN patients; p 1-3, comparison of OP-OA patients; p 2-3, comparison of OPN- OA patients; Q angle, quadrieps angle; R, right; VAS, Visual Analog Scale. Bold p values indicate comparisons with statistically significant differences (p<.05) between groups.

**Table 4.** Comparison of Muscle Strength Between Groups

	OP Group (n = 22) median [Q1;Q3]	OPN Group (n = 22) median [Q1;Q3]	OA Group (n = 22) median [Q1;Q3]	*P*
Hip Flex-R	5.00 [5.00; 5.00]	5.00 [5.00; 5.00]	5.00 [5.00; 5.00]	.869
Hip Flex-L	5.00 [5.00; 5.00]	5.00 [5.00; 5.00]	5.00 [4.00; 5.00]	.122
Hip Ext-R	5.00 [5.00; 5.00]	5.00 [5.00; 5.00]	5.00 [5.00; 5.00]	.571
Hip Ext-L	5.00 [5.00; 5.00]	5.00 [5.00; 5.00]	5.00 [5.00; 5.00]	.684
Hip Abd-R	5.00 [5.00; 5.00]	5.00 [5.00; 5.00]	5.00 [5.00; 5.00]	.582
Hip Abd-L	5.00 [5.00; 5.00]	5.00 [5.00; 5.00]	5.00 [5.00; 5.00]	.769
Hip Add-R	5.00 [4.75; 5.00]	5.00 [4.75; 5.00]	5.00 [5.00; 5.00]	.915
Hip Add-L	5.00 [4.75; 5.00]	5.00 [4.75; 5.00]	5.00 [5.00; 5.00]	.915
Hip E.Rot-R	5.00 [5.00; 5.00]	5.00 [5.00; 5.00]	5.00 [5.00; 5.00]	.898
Hip E.Rot-L	5.00 [5.00; 5.00]	5.00 [5.00; 5.00]	5.00 [5.00; 5.00]	1
Hip I.Rot-R	5.00 [5.00; 5.00]	5.00 [5.00; 5.00]	5.00 [5.00; 5.00]	.571
Hip I.Rot-L	5.00 [5.00; 5.00]	5.00 [5.00; 5.00]	5.00 [5.00; 5.00]	.684
Knee Flex-R	5.00 [5.00; 5.00]	5.00 [5.00; 5.00]	5.00 [5.00; 5.00]	.769
Knee Flex-L	5.00 [5.00; 5.00]	5.00 [5.00; 5.00]	5.00 [5.00; 5.00]	.854
Knee Ext-R	5.00 [5.00; 5.00]	5.00 [5.00; 5.00]	5.00 [5.00; 5.00]	.582
Knee Ext-L	5.00 [5.00; 5.00]	5.00 [5.00; 5.00]	5.00 [5.00; 5.00]	.769
Foot P.Flex-R	5.00 [5.00; 5.00]	5.00 [5.00; 5.00]	5.00 [5.00; 5.00]	.808
Foot P.Flex-L	5.00 [5.00; 5.00]	5.00 [5.00; 5.00]	5.00 [5.00; 5.00]	.769
Foot D.Flex-R	5.00 [5.00; 5.00]	5.00 [5.00; 5.00]	5.00 [5.00; 5.00]	.582
Foot D.Flex-L	5.00 [5.00; 5.00]	5.00 [5.00; 5.00]	5.00 [5.00; 5.00]	.582
Foot Inv.-R	5.00 [5.00; 5.00]	5.00 [5.00; 5.00]	5.00 [5.00; 5.00]	.854
Foot Inv.-L	5.00 [5.00; 5.00]	5.00 [5.00; 5.00]	5.00 [5.00; 5.00]	.582
Foot Ev.-R	5.00 [5.00; 5.00]	5.00 [5.00; 5.00]	5.00 [5.00; 5.00]	.375
Foot Ev.-L	5.00 [5.00; 5.00]	5.00 [5.00; 5.00]	5.00 [5.00; 5.00]	.869

Abd, abduction; Add, addition; D. Flex, dorsi flexion; E. Rot, external rotation; Ev, eversiyon; Ext, extansion; Flex, flexion; I. Rot, internal rotation; Inv, inversion; L, left; OA, osteoarthritis; OP, osteoporosis; OPN, osteopenia; P. Flex, plantar flexion; R, right.

**Table 5.** Osteoporosis, Osteopenia and Osteoarthritis Correlation Analysis (Right) (r and *p* Values)

		OP Group					OPN Group					OA Group				
		Q Angle-R	NDT-R	FPI-R	VAS	BMI	Q Angle-R	NDT-R	FPI-R	VAS	BMI	Q Angle-R	NDT-R	FPI-R	VAS	BMI
Q Angle-R	r	1.00	**−0.91**	**−0.94**	0.30	0.46	1.00	**−0.89**	**−0.84**	0.12	−0.26	1.00	**−0.68**	**−0.82**	0.23	−0.11
	*p*	.	**<.01**	<.01*	.16	.02	.	**<.01**	<.01*	.34	.22	.	<.01*	<.01*	.30	.62
NDT-R	r		1.00	**0.90**	−0.34	−0.34		1.00	**0.69**	-0.29	0.10		1.00	**0.85**	-0.45	−0.05
	*p*		.	<.01*	.11	.11		.	<.01*	.17	.63		.	<.01*	.35	.81
FPI-R	r			1.00	−0.44	−0.36			1.00	0.04	0.20			1.00	−0.41	−0.16
	*p*			.	.04	.09			.	.83	.35			.	.05	.47
VAS	r				1.00	0.24				1.00	0.42				1.00	−0.14
	*P*				.	.27				.	.04				.	.51
BMI	r					1.00					1.00					1.00
	*p*					.					.					.

BMI, body mass index; FPI, Foot Posture Index; NDT, Navicular Drop Test; OA, osteoarthritis; OP, osteoporosis; OPN, osteopenia; Q angle, quadrieps angle; R, right; VAS, Visual Analog Scale. *Significant at .05 level. Bold correlation coefficients (r) and p values represent statistically significant and strong correlations.

**Table 6.** Osteoporosis, Osteopenia and Osteoarthritis Correlation Analysis (Left) (r and *P* Values)

		OP Group					OPN Group					OA Group				
		Q Angle-L	NDT-L	FPI-L	VAS	BMI	Q Angle-L	NDT-L	FPI-L	VAS	BMI	Q Angle-L	NDT-L	FPI-L	VAS	BMI
Q Angle-L	r	1.00	**−0.91**	**−0.96**	0.46	0.45	1.00	**−0.88**	**−0.93**	0.07	0.02	1.00	**−0.89**	**−0.84**	0.21	0.22
	*P*	.	**<.01**	**<.01**	.02	.03	.	**<.01**	**<.01**	.73	.92	.	**<.01**	**<.01**	.34	.31
NDT-L	r		1.00	**0.87**	**−0.59**	−0.32		1.00	**0.75**	−0.10	−0.07		1.00	**0.70**	−0.19	−0.33
	*P*		.	**<.01**	**<.01**	.14		.	**<.01**	.63	.74		.	**<.01**	.38	.13
FPI-L	r			1.00	−0.44	−0.47			1.00	0.05	−0.01			1.00	−0.05	−0.26
	*P*			.	.04	.02			.	.80	.96			.	.81	.23
VAS	r				1.00	0.24				1.00	0.42				1.00	−0.14
	*P*				.	.27				.	.04				.	.51
BMI	r					1.00					1.00					1.00
	*P*					.					.					.

BMI, body mass index; FPI, Foot Posture Index; L, left; NDT, Navicular Drop Test; OA, osteoarthritis; OP, osteoporosis; OPN, osteopenia; Q angle, quadrieps angle; VAS, Visual Analog Scale. Bold correlation coefficients (r) and p values represent statistically significant and strong correlations.

## Data Availability

The data that support the findings of this study are available on request from the corresponding author.
